# Quercetin, a Promising Clinical Candidate for The Prevention of Contrast-Induced Nephropathy

**DOI:** 10.3390/ijms20194961

**Published:** 2019-10-08

**Authors:** Laura Vicente-Vicente, David González-Calle, Alfredo Ginés Casanova, María Teresa Hernández-Sánchez, Marta Prieto, Juan Carlos Rama-Merchán, Javier Martín-Moreiras, Francisco Martín-Herrero, Pedro Luis Sánchez, Francisco J. López-Hernández, Ignacio Cruz-González, Ana Isabel Morales

**Affiliations:** 1 Toxicology Unit, University of Salamanca, 37007 Salamanca, Spain; lauravicente@usal.es (L.V.-V.); alfredogcp@usal.es (A.G.C.); hsteresa@usal.es (M.T.H.-S.); martapv@usal.es (M.P.); flopezher@usal.es (F.J.L.-H.); 2 Institute of Biomedical Research of Salamanca (IBSAL), 37007 Salamanca, Spain; jmmoreiras@gmail.com (J.M.-M.); fmartinherrero@usal.es (F.M.-H.); pedrolsanchez@secardiologia.es (P.L.S.); cruzgonzalez.ignacio@gmail (I.C.-G.); 3 Group of Translational Research on Renal and Cardiovascular Diseases (TRECARD), 37007 Salamanca, Spain; 4 Department of Cardiology, Hospital Universitario de Salamanca, 37007 Salamanca, Spain; davidcalle1990@hotmail.com (D.G.-C.); ramamerchan@hotmail.com (J.C.R.-M.)

**Keywords:** quercetin, glomerular injury, contrast media, contrast-induced nephropathy, prevention, flavonoids, albuminuria

## Abstract

Iodinated contrast media (CM) are the leading cause of acute renal failure of toxic origin. Between 21% and 50% of patients that receive them develop contrast-induced nephropathy (CIN). All prophylactic measures used so far have failed to provide effective prevention. Since oxidative stress is involved in the damage, a possible preventive strategy could be the administration of antioxidant substances, such as quercetin. This compound has shown renoprotective effects in experimental studies. The aim of this study was to evaluate whether quercetin may be helpful in preventing CIN in patients undergoing coronary catheterization. A clinical phase II study was conducted. Patients were distributed in two groups, namely, CM (patients who only received contrast media) and CM+Q (patients who were pretreated with quercetin orally for 3–5 days). Results showed less incidence of CIN in the CM+Q group, possibly due to glomerular protection, evidenced by a lower increase in serum creatinine and albuminuria; and a lower decrease in the glomerular filtration rate (GFR). Furthermore, in this group, the relative risk of developing CIN observed in patients that received a high dose of contrast media was inferior. In conclusion, this is the first study that demonstrates that quercetin is a promising safe candidate in preventing CIN.

## 1. Introduction

Contrast-induced nephropathy (CIN) is the major complication related to cardiac diagnostic or interventional procedures that require the use of contrast media (CM). The European Society of Urogenital Radiology defines CIN as an increase in serum creatinine of ≥0.5 mg/dL or ≥25% from the baseline value within three days after CM administration, in the absence of other causes for impaired renal function [1]. CIN is the third most common cause of acute renal failure acquired in the hospital [2] and it is associated with significant morbidity and mortality in the short and long term [3,4]. This condition has also been linked to longer in-hospital stay and higher incidence of chronic renal failure [5].

CIN pathogenesis is not fully understood and seems to be due to different factors. The intravenous injection of CM induces an initial increase in the glomerular filtration rate (GFR) due to a transient afferent arteriolar vasodilation. Subsequently, a prolonged vasoconstriction occurs, leading to a sustained decrease in the GFR over time, whereby a renal ischemia occurs. This state of hypoxia results in the formation of reactive oxygen species that can produce tubular damage and further intensify the reduction of the GFR. Because CM are excreted through the kidney, renal tubular cells are directly exposed to the toxicant. That is why, in preclinical studies, massive intratubular necrosis and tubular obstruction have been observed [6].

Only two procedures have shown a relative efficacy in preventing CIN in the clinical setting—the reduction of CM volume and isotonic hydration prior to catheterization. Other approaches such as the administration of sodium bicarbonate [7] or diuretics [8] have also been tested, without effective results. Thus, these measures are not enough, and a large number of patients continue developing CIN. For this reason, new strategies to reduce CIN incidence and severity are being pursued. Specifically, animal studies are testing nephroprotective substances designed to either facilitate CM removal in order to prevent their accumulation in renal tubules, to inhibit tubular CM uptake, or to form less toxic complexes, or to modulate signaling pathways involved in their toxic effect. However, these compounds are still in preclinical stages and the effect on humans is unknown [9,10,11]. A possible solution to this problem could be the administration of antioxidants since, as previously mentioned, the formation of free radicals seems to play an important role in the pathogenesis of renal damage caused by CM. An antioxidant candidate as nephroprotector could be quercetin. This compound is an abundant vegetal flavonoid in the Mediterranean diet to which several properties have been attributed, such as antioxidant, anti-inflammatory, analgesic, antihistamine, cholesterol reducer, antiviral, and renal hemodynamic modulator (reviewed in Vicente-Vicente et al., 2013 [12]). In addition, preclinical studies have been published supporting the conclusion that quercetin exerts protection against renal damage induced by cadmium chronically administered, due to its properties as antioxidant, anti-inflammatory, vasodilator, and metallothionein inductor [13,14]. It has also proven to be effective in preventing nephrotoxicity caused by the antineoplastic cisplatin [15]. Its renoprotective effect has also been evaluated in vitro [16]. In tubular epithelial cells, quercetin was linked to decreased lipid peroxidation and free radical removal. It was also tested as a nephroprotector in an ischemia/reperfusion model, in which the increased nitric oxide concentration was reduced by quercetin pretreatment [17]. 

Regarding quercetin safety in humans, a large number of clinical studies have been conducted both in phase I and phase II, testing several doses of quercetin [18,19,20,21,22,23,24,25,26,27,28,29]. Only in one clinical trial have some adverse effects associated with this product been reported. The intravenous administration of a high dose (51.3 mg/kg) caused dyspnea, emesis, and nephrotoxicity in one patient [25]. None of the studies in which quercetin was administered orally has reported adverse effects, indicating that quercetin is safe for human administration [17,18,19,21,23,24,26,27,28], even at doses of 1500 mg/day for 84 days [20].

With the arguments above, quercetin can be considered as a promising candidate as nephroprotectant against CIN. Therefore, the objectives of this work were to study the effectiveness of the flavonoid quercetin as a novel strategy to reduce the incidence of CIN in cardiac patients and to evaluate its possible renal protective effect through the determination of tubular and glomerular damage biomarkers.

## 2. Results

### 2.1. Patient Characteristics

During the recruitment period, 192 patients agreed to participate in the study, of which 134 were included in the CM group and 58 in the CM+Q group. Sample collection was done in two phases; first, control (CM group) samples were collected and, second, the samples of patients receiving quercetin (CM+Q) were collected. Although the collection period was similar in both groups, fewer patients agreed to take quercetin. For this reason, the number of patients was different between the groups. Despite this, anthropometric characteristics and risk factors were homogeneous in both groups (Table 1). Regarding pharmacological treatment in each group, no differences were observed between the two groups. The most commonly consumed drugs were those related to the pathologies mentioned as risk factor (i.e., antihypertensives, cholesterol-lowering drugs, and antidiabetics. Since patients had cardiac pathologies, it was also observed that almost half of the patients in each group consumed antiplatelet agents (acetyl salicylic acid (ASA) and others). It is remarkable that a high percentage of patients in the CM+Q group (17.0%) consumed non-steroidal anti-inflammatory drugs (NSAIDs), whereas in the CM group only 7.3% consumed them (Table 2).

### 2.2. Basal Renal Function

The baseline serum creatinine, urea, and the GFR were similar in both groups as shown in Table 3 (*p* > 0.05).

### 2.3. Quercetin Efficacy as Nephroprotector

**Figure 1:**
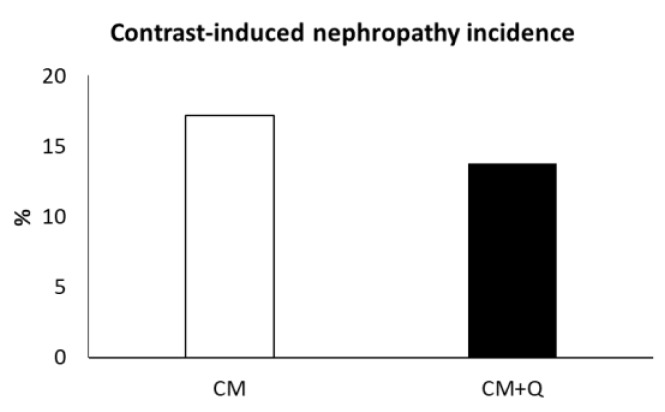
Contrast-induced nephropathy (CIN) incidence, calculated as % of patients whose increase in serum creatinine during the three days post contrast media was higher than 0.5 mg/mL or 25% with respect to its basal value. No significant differences were found (*p* > 0.05).

First, CIN incidence was calculated in each group (Figure 1), by calculating the percentage of patients whose creatinine increase was greater than 0.5 mg/dL or 25% from the baseline value. The CIN incidence was lower in the CM+Q group (13.8% in CM+Q versus 17.2% in CM).

**Figure 2:**
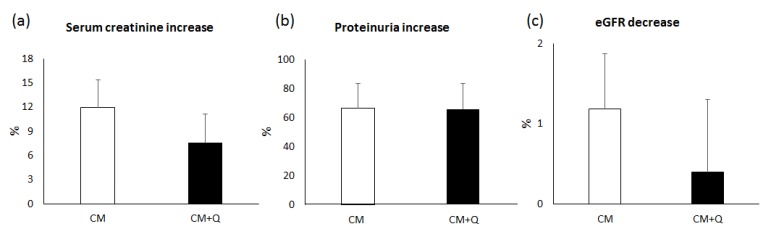
Renal function assessment in each study group. CM, patients who were administered iodinated contrast media; CM+Q, patients receiving iodinated contrast media and quercetin. (**a**) Increase in serum creatinine; (**b**) Increase in proteinuria; (**c**) Decrease in eGFR calculated using the Chronic Kidney Disease Epidemiology Collaboration (CKD-EPI) formula. Increases or decreases were calculated as % using basal biomarker data and the highest value observed during the 72 h after contrast media administration. Data are expressed as mean ± standard error of the mean. No significant differences were found (*p* > 0.05). eGFR, estimated glomerular filtration rate

**Figure 3:**
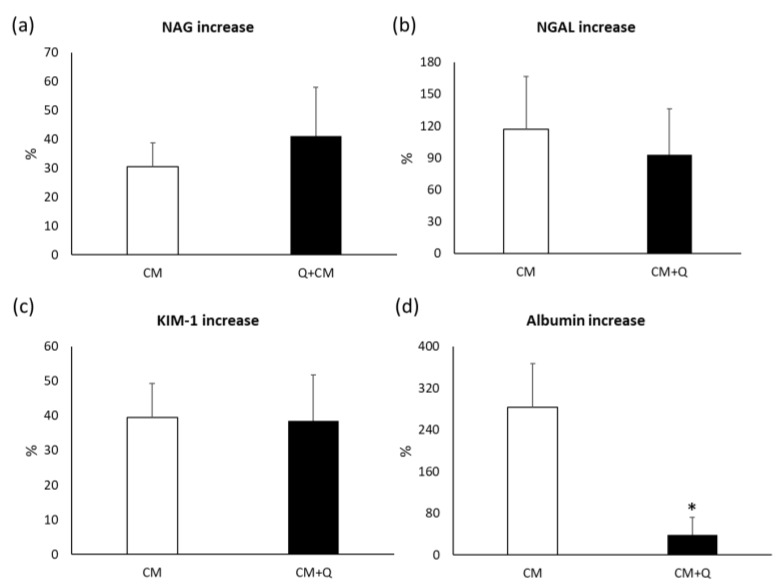
Quantification of early kidney damage biomarkers in each study group. CM, patients who were administered iodinated contrast media; CM+Q, patients receiving iodinated contrast media and quercetin. (**a**) Increase in urinary NAG; (**b**) Increase in urinary NGAL; (**c**) Increase in urinary KIM-1; (**d**) Increase in urinary albumin. Increase was calculated as % using basal biomarker data and the highest value observed during the 72 h after contrast media administration. Data are expressed as mean ± standard error of the mean. * *p* < 0.05 vs. CM group. KIM-1, kidney injury molecule 1; NAG, N-acetyl-β-D-glucosaminidase; NGAL, neutrophil gelatinase-associated lipocalin.

Subsequently and with the aim of assessing the ability of quercetin to prevent kidney injury caused by exposure to CM, the increases in serum creatinine (Figure 2a), proteinuria (Figure 2b), urinary N-acetyl-β-D-glucosaminidase (NAG; Figure 3a), urinary neutrophil gelatinase-associated lipocalin (NGAL; Figure 3b), urinary kidney injury molecule 1 (KIM-1; Figure 3c), and albuminuria (Figure 3d) were calculated, as well as the decrease in the glomerular filtration rate (GFR; Figure 2c) using the Chronic Kidney Disease Epidemiology Collaboration (CKD-EPI) formula. A lower increase in serum creatinine concentration and albuminuria was observed in patients treated with quercetin when compared with untreated patients (serum creatinine increase: 7.62% ± 3.49% in CM+Q versus 11.91% ± 3.43% in CM, *p*-value = 0.97; albuminuria increase: 38.3% ± 33.5% in CM+Q versus 282.9% ± 84.0% in CM, *p*-value = 0.02). No differences in proteinuria, NAG, NGAL, and KIM-1 were observed between groups (*p* > 0.05). However, the GFR showed a greater decrease in patients who did not receive quercetin, which is consistent with the results of plasma creatinine.

### 2.4. Relative Risk of CIN from Each Risk Factor

In each study group, the relative risk (RR) of developing CIN was calculated for each parameter considered as a risk factor (Table 4). RR indicates the probability of an event occurring (in this study, the event is CIN) by comparing the number of patients that present the risk factor (arterial hypertension, dyslipidemia, etc.) with those who do not present it. A higher value indicates a higher probability of developing CIN because of the risk factor. Values were very similar in both groups although slightly lower in the CM+Q group, except for the RR calculated with the volume of CM. Administration of more than 350 mL was associated with a greater number of CIN cases (more than twice) in patients in the CM group than in patients that received quercetin.

### 2.5. Quercetin Safety

The flavonoid administration at a dose of 500 mg every 8 h for 3–5 days produced no adverse effects, as shown by the results of the surveys conducted in quercetin-treated group, in which only 3% of patients reported moderate gastrointestinal pain.

## 3. Discussion

CM administration has increased in recent years [30]. This fact, along with the elevated prevalence of CIN risk factors (chronic kidney disease and diabetes mellitus), represents an immediate urgency in the search for strategies that can prevent the development of kidney damage following CM administration.

It is known that oxidative stress plays an important role in the pathogenesis of CM-induced renal damage [6]. In this context, this study proposed the prophylactic treatment with the antioxidant quercetin as a possible solution.

Our data demonstrate that the administration of quercetin is able to attenuate the increase in albumin excretion. If albuminuria truly reflects a malfunction in the glomerular filtration barrier [31], then this study lends credence to the hypothesis that quercetin may be protective through its biological effect ameliorating oxidative damage at the level of the glomerular apparatus [32]. This finding has even been confirmed in studies with other antioxidants such as N-acetyl-cysteine against CIN [33]. It should also be noted that the protective capacity of quercetin is evident even though the percentage of smokers was higher in the CM+Q group. Since tobacco contributes to oxidative stress generation, it is possible that if the prevalence of this risk factor had been similar in both groups, the observed nephroprotective effect would have been greater. 

Oxidative stress protection could justify the lower increase in serum creatinine and the lower incidence of CIN in patients who were treated with quercetin. Regarding creatinine, the lack of statistical significance in this parameter could be because creatinine measurement is too insensitive to detect minor insults to the glomeruli. On the other hand, albuminuria has a greater capacity to detect kidney damage in initial stages [34], increasing its urine excretion earlier and more specifically than serum creatinine, which would explain the higher difference. Regarding the incidence of CIN, it was 17.2% in the patients who only received the CM, in agreement with data reported for patients with heart disease and similar risk factors shown by our study group [2,35,36]. The incidence was lower (13.8%) in the CM+Q group. The calculation of the GFR using the CKD-EPI formula showed a greater decrease in patients who did not receive quercetin, which is consistent with the results described above. 

In order to evaluate the possible protective effect of quercetin on contrast-induced tubular damage, the excretion of some specific markers such as NAG, NGAL, and KIM-1 was measured. NAG is an enzyme which is released to the urine after an injury in the tubular cells. Clinical studies have shown that its urinary excretion increase in the context of a variety of drugs, including contrast media, occurs earlier than the increase in serum creatinine [37,38,39]. NGAL is a protein whose expression in the kidney is induced as a consequence of hypoxic damage, especially in proximal tubular cells [40]. Previous studies have shown a significant increase of this protein in urine 2 h after CM administration [41]. On the other hand, KIM-1 is a protein that confers to cells the ability to phagocytose dead cells after an ischemic injury of the kidney [42]. Its utility to diagnose CIN has not been widely studied. In accordance with these studies, our results show that CM exert tubular toxicity, although quercetin appears to have no capacity to protect at this level, since the increase is similar in both groups studied (Figure 3). 

The definition of CIN determines that renal damage occurs as a consequence of CM administration. It is necessary to take into account that the presence of other risk factors (hypertension, diabetes, consumption of NSAIDs, etc.) can increase the risk of suffering CIN; therefore, the RR for CIN because of each risk factor was calculated. The value of RR to suffer CIN was similar in all evaluated risk factors in both groups, indicating that quercetin does not influence any of these parameters. However, the RR calculated in patients whose CM volume is greater than 350 mL showed that the risk of CIN due to the administration of high amounts of CM is lower in patients receiving quercetin. These data are very important and suggest that quercetin may reduce the risk of CIN derived from CM administration, allowing the management of larger volumes of CM without increasing the risk of CIN, which may be necessary in diagnostic or surgical interventions.

Other data collected during the study were the days of hospital stay and mortality. In both groups, the mean number of days admitted to the hospital was 6. In terms of mortality, 14.7% of patients in the CM group died some days after CM administration, whereas in CM+Q this number decreased to 7.8%. Although it is a very important difference, it cannot be completely attributed to flavonoid administration since it may be due to other factors related to the pathology according to which the patient was operated. Even so, the reduction of CIN by quercetin could be involved in this lower number of deaths, but confirming the hypothesis would require a greater number of patients. 

On the other hand, no toxic effects were associated with the use of quercetin, except for abdominal pain which was reported by 3% of patients treated with this substance. With these data, it could be argued that the oral administration of quercetin at a dose of 1500 mg/day is safe in cardiac patients with associated risk factors. This result is in agreement with the information contained in the literature, where the oral administration of quercetin was safe, both in the healthy population [18,19,24] and in patients with different diseases such as gastrointestinal diseases [28] or cardiovascular diseases, hypertension, metabolic syndrome, and high cholesterolemia [20,21,43,44].

Considering all the above, these results suggest a potential effectiveness of quercetin as nephroprotector against CM-induced renal damage with absence of adverse effects. However, some limitations of this study make it difficult to repeat in humans the results obtained in preclinical studies [13,14,15]. The main limitation was the poor oral bioavailability of flavonoids [45]. In laboratory animals, the i.p. mode was used, which allows the administration of the highest doses of quercetin in comparison to the oral mode used in patients. Moreover, the present data cannot be compared with previous works since this is the first study designed to assess the use of quercetin as a human nephroprotective substance. It is possible that if the bioavailability of quercetin increases, a greater protection will be provided against renal damage produced by CM. The study of new formulations of quercetin with greater absorption could be a good solution. Other limitations that may have affected the study are, first, that the CM group did not receive the oral formulation without quercetin, so the placebo effect was not contemplated. However, in this work, it was considered that this did not interfere since the efficacy was evaluated through objective biochemical parameters. Second, it would have been of special interest to know the blood levels of quercetin in each patient in order to associate the flavonoid concentration with the observed protective effect. However, the necessary techniques to carry this out were not available.

This study suggests that quercetin may attenuate glomerular injury in patients who received CM and were pretreated with this flavonoid. The administration of quercetin appears to be a promising candidate for the prevention of CIN. Thus, this study is a first possible step for including quercetin as a prophylactic measure in CIN management protocols. 

## 4. Materials and Methods 

### 4.1. Study Population

The experimental protocol was approved by the “Ethics Committee of research with medicines of Salamanca” of the “Complejo Asistencial Universitario de Salamanca” (Salamanca, Spain), reference number PI 2011 03 001 (2 May 2011). All the following procedures were performed in accordance with the ethical standards of the responsible committee on human experimentation (institutional and national) and with the Helsinki Declaration of 1975, as revised in 2000. Informed consent was obtained from all patients included in the study.

In this randomized prospective study, patients suffering from acute coronary syndrome without ST elevation who would receive iodinated CM for diagnostic procedures were enrolled. It was estimated that, in order to obtain results that were statistically relevant, at least 50 patients treated with quercetin and their respective controls (patients with the same conditions but without quercetin) would be required. Patients who agreed to participate met the inclusion criteria (be over 18 years old and sign an informed consent). Two experimental groups were made, namely, Group CM (patients who only received CM) and Group CM+Q (patients who were pretreated with quercetin orally (500 mg/8 h)). The planned posology was to start quercetin administration two days before CM administration and continue the following two days, but due to the urgency of some catheterizations the total posology of quercetin ranged between 3 and5 days.

### 4.2. Samples and Clinical Data Collection

Blood and urine samples were collected immediately before CM administration (basal) and 24, 48, and 72 h after CM. These samples were centrifuged and stored at −80 °C.

For each participant, the following data were collected: age, gender, body mass index, risk factors associated with CIN (diabetes mellitus, arterial hypertension, and smoking), CM data (CM administered and volume), and pharmacological treatment received before/during CM administration. 

### 4.3. Renal Function Evaluation

Renal function was determined at time points 0, 24, 48, and 72 h by quantifying serum creatinine and urea concentrations, proteinuria, GFR, and urinary NAG, NGAL, KIM-1, and albumin excretion. The increase (in percentage) of each parameter studied was calculated using the following formula:

Biomarker increase (%) = (Maximum value − Basal value)/(Basal value) × 100(1)

where Maximum value means the highest value reached by the parameter during the first 72 h after CM, whereas Basal value means the value of the biomarker before CM administration.

Serum creatinine was evaluated using an automatic analyzer (Roche/Hitachi 917; Mannheim, Germany). It was considered that the patient suffered CIN if creatinine increased >25% of its basal value in the first 72 h after CM administration [1]. The same analyzer was used to measure serum urea. The CKD-EPI formula [46] was used to estimate the GFR. 

Urinary excretion of proteins was determined by the Bradford colorimetric method [47]. Urinary NAG activity was measured using a commercial kit (“N-Acetyl-β-D-glucosaminidase NAG assay kit DZ062A-K”, Diazyme, Poway, CA, USA), following the manufacturer’s instructions. Urinary levels of NGAL, KIM-1, and albumin were measured by commercial ELISAs (“Human NGAL ELISA Kit 036CE”, BioPorto Diagnostics, Hellerup, Denmark; “Human KIM-1 ELISA Kit #ADI-900-226”, Enzo Life Sciences, Farmingdale, NY, USA; and “Human Albumin ELISA Kit E88-129”, Bethyl Laboratories, Montgomery, TX, USA, respectively), according to the manufacturer’s instructions. All urinary biomarkers quantified were corrected by the concentration of creatinine in urine.

### 4.4. Statistical Analysis

Results are presented as mean ± standard error of the mean (SEM). Data were statistically analyzed using the IBM SPSS Statistics 20 software (Chicago, IL, USA). Because the results were not normally distributed (according to the Kolmogorov–Smirnov test), they were analyzed using the nonparametric Mann–Whitney test in order to check statistically significant differences between groups. To compare qualitative data, a Pearson’s chi-squared test was applied to evaluate the homogeneity between groups. It was considered a significant difference when *p* < 0.05.

### 4.5. Relative Risk (RR) of CIN Calculation

For each risk factor (diabetes mellitus, dyslipidemia, arterial hypertension, and smoking), the RR of developing CIN was calculated in both groups, with the aim of studying if quercetin administration reduced or not the probability to develop CIN due to the risk factor. In addition, the volume of CM administered was considered as another risk factor in several studies [48,49,50]. For this reason, in each group, patients were separated into two groups, one for those who were given less than 350 mL and another for those who were given 350 mL or more.

RR (with its 95% confidence interval) was calculated for each risk factor using the following formula:

RR = (a/(a + b))/(c/(c + d)),(2)

where *a* is the number of patients that develop CIN and have the risk factor; *b* is the number of patients without CIN but with the risk factor; *c* is the number of patients that develop CIN but do not have the risk factor; and *d* is the number of patients without CIN nor risk factor.

The statistical significance was inferred at a two-sided *p*-value < 0.05.

### 4.6. Quercetin Safety

To ensure the safety of quercetin, the nursing service was responsible for monitoring the health of patients during and after treatment with quercetin. In addition, a questionnaire about adverse effects associated with the consumption of this flavonoid was distributed. Specifically, patients were questioned about headaches, palpitations, diarrhea, fatigue, edema, nausea or vomiting, abdominal pain, dizziness, and fever.

## Figures and Tables

**Table 1:** Anthropometric characteristics, risk factors associated with contrast media-induced nephropathy of patients in each study group, and information related to the contrast agent administered (for each type, percentage of patients, and volume). CM, patients who were administered iodinated contrast media; CM+Q, patients pretreated with quercetin. * *p* < 0.05 vs. CM group. BMI, body mass index; SEM, standard error of the mean.

	Group	
	CM Group	CM+Q Group
Anthropometric Characteristics		
Men (%)	77.6	67.3
Women (%)	22.4	32.7
Age (years; mean ± SEM) (minimum-maximum)	71.4 ± 1.1 (39–91)	68.5 ± 1.4 (46–88)
BMI (mean ± SEM)	27.6 ± 0.4	31.8 ± 3.7
Risk Factors		
Diabetes mellitus (%)	27.6	31.0
Dyslipidemia (%)	44.0	51.7
Arterial hypertension (%)	56.7	53.4
Smoking (%)	20.1	36.2 *
Type of Contrast Agent		
CM administered (%)/		
Volume mL (mean ± SEM)		
Iodixanol	70.1/ 295.0 ± 15.0	63.8/ 274.1 ± 18.7
Iohexol	22.4/ 260.0 ± 21.7	25.9/ 308.4 ± 28.0
Iodine (not specified)	0.8/ 100.0 ± 0.0	3.5/ 600.0 ± 0.0
Without data	6.7	5.2

**Table 2:** Drug consumption before contrast media administration. ACE, angiotensin-converting enzyme; ARBs, angiotensin II receptor blockers; ASA, acetyl salicylic acid; NSAIDs, non-steroidal anti-inflammatory drugs. Data are expressed as percentage of patients. No statistical differences were observed between the groups.

Pharmacological Group	CM Group	CM+Q Group
Hypertension Treatment		
ACE inhibitors	26.9	27.6
Aldosterone receptor antagonists	0.8	2.12
Alpha blockers	4.1	10.6
ARBs	15.4	17.0
Beta blockers	26.9	19.1
Calcium channels antagonists	17.9	4.2
Diuretics	28.5	23.4
Dyslipidemia Treatment		
Statins	38.2	31.9
Diabetes Mellitus Treatment		
Antidiabetics	21.1	21.3
Thrombus Prevention Treatment		
ASA	35.8	31.9
Antiplatelet agents	25.2	12.8
Others		
NSAIDs	7.3	17.0

**Table 3:** Basal renal function in both groups. Crs, serum creatinine; eGFR, estimated glomerular filtration rate; CKD-EPI, Chronic Kidney Disease Epidemiology Collaboration. Data are expressed as mean ± standard error of the mean. No significant differences were found (*p* > 0.05).

	CM Group	CM+Q Group
Crs (mg/dL)	1.08 ± 0.04	0.96 ± 0.04
Serum urea (mg/dL)	52.20 ± 2.54	48.91 ± 3.00
eGFR CKD-EPI (mL/min/1.73 m^2^)	82.61 ± 1.09	85.93 ± 1.44

**Table 4:** Calculation of the relative risk to develop contrast-induced nephropathy for each risk factor. RR, relative risk; CM, patients who were administered iodinated contrast media; CM+Q, patients pretreated with quercetin before CM administration. * Statistically significant (*p* < 0.05).

Risk Factor	CM			CM+Q		
	RR	95% Confidence Interval		RR	95% Confidence Interval	
		Lower Limit	Upper Limit		Lower Limit	Upper Limit
Arterial hypertension	1.19	0.83	1.68	0.87	0.41	3.85
Diabetes mellitus	1.15	0.57	2.29	0.74	0.21	2.63
Dyslipidemia	0.73	0.41	1.32	1.56	0.85	2.82
Smoking	1.17	0.50	2.71	1.06	0.40	2.78
CM volume >350 mL	1.83 *	1.07	3.14	0.80	0.22	2.86

## Abbreviations

ACE	Angiotensin converting enzyme
ARBs	Angiotensin II receptor blockers
ASA	Acetyl salicylic acid
BMI	Body mass index
CIN	Contrast-induced nephropathy
CKD-EPI	Chronic Kidney Disease Epidemiology Collaboration
CM	Contrast media
Crs	Serum creatinine
eGFR	Estimated glomerular filtration rate
GFR	Glomerular filtration rate
KIM-1	Kidney injury molecule 1
NAG	N-acetyl-β-D-glucosaminidase
NGAL	Neutrophil gelatinase-associated lipocalin
NSAIDs	Nonsteroidal anti-inflammatory drugs
Q	Quercetin
RR	Relative risk
SEM	Standard error of the mean
